# Diagnostic Accuracy of the Artificial Intelligence Methods in Medical Imaging for Pulmonary Tuberculosis: A Systematic Review and Meta-Analysis

**DOI:** 10.3390/jcm12010303

**Published:** 2022-12-30

**Authors:** Yuejuan Zhan, Yuqi Wang, Wendi Zhang, Binwu Ying, Chengdi Wang

**Affiliations:** 1 Department of Respiratory and Critical Care Medicine, West China Medical School/West China Hospital, Sichuan University, Chengdu 610041, China; 2 Department of Laboratory Medicine, West China Medical School/West China Hospital, Sichuan University, Chengdu 610041, China

**Keywords:** pulmonary tuberculosis, artificial intelligence, medical imaging, diagnostic accuracy, sensitivity, specificity

## Abstract

Tuberculosis (TB) remains one of the leading causes of death among infectious diseases worldwide. Early screening and diagnosis of pulmonary tuberculosis (PTB) is crucial in TB control, and tend to benefit from artificial intelligence. Here, we aimed to evaluate the diagnostic efficacy of a variety of artificial intelligence methods in medical imaging for PTB. We searched MEDLINE and Embase with the OVID platform to identify trials published update to November 2022 that evaluated the effectiveness of artificial-intelligence-based software in medical imaging of patients with PTB. After data extraction, the quality of studies was assessed using quality assessment of diagnostic accuracy studies 2 (QUADAS-2). Pooled sensitivity and specificity were estimated using a bivariate random-effects model. In total, 3987 references were initially identified and 61 studies were finally included, covering a wide range of 124,959 individuals. The pooled sensitivity and the specificity were 91% (95% confidence interval (CI), 89–93%) and 65% (54–75%), respectively, in clinical trials, and 94% (89–96%) and 95% (91–97%), respectively, in model-development studies. These findings have demonstrated that artificial-intelligence-based software could serve as an accurate tool to diagnose PTB in medical imaging. However, standardized reporting guidance regarding AI-specific trials and multicenter clinical trials is urgently needed to truly transform this cutting-edge technology into clinical practice.

## 1. Introduction

Tuberculosis (TB) is one of the major communicable diseases that seriously endanger human health primarily in developing countries [1], and at least 5.8 million people were estimated to have contracted tuberculosis in 2020. However, around one-sixth of people with active tuberculosis are left undetected or not officially reported each year, which may delay the progress of elimination of this disease before 2035 [2]. Timely diagnosis and treatment could benefit a wide range of tuberculosis patients and minimize the transmission of pathogen in the whole population.

Mycobacterium tuberculosis culture on solid and/or liquid media is still the golden standard for diagnosis. However, the efficiency of culture-based diagnosis in clinical practice is diminished due to long turnaround times and lack of laboratory infrastructure, especially in resource-limited countries. To solve this, the Xpert MTB/RIF assay has emerged as a maturely implemented tool in many countries haunted greatly by TB disease, which is a semiautomated rapid molecular method allowing for rapid diagnosis based upon detection of Mycobacterium tuberculosis DNA and resistance to rifampicin [3], but the application of such rapid tests remains far too limited, with only 1.9 million (33%) people having taken it as an initial diagnostic test in 2022. Simultaneously, the World Health Organization (WHO) has recommended using chest X-ray (CXR) images as a screening technique to better target individuals needing a further microbiological test, which has been proved to be relatively easy to operate, low-cost and highly sensitive [4]. However, an accurate diagnosis with CXRs extremely depends on the clinical experience of radiologists, which poses a huge challenge in the aforementioned countries. As such, there has been increasing interest in using artificial-intelligence-based (AI-based) software in medical imaging for pulmonary tuberculosis (PTB) detection, achieving diagnostic accuracy improvement and cost reduction at the same time. Currently, more than 40 AI-based software programs certified for CXR or computed tomography (CT) examination are available, among which only five are certified for CXR tuberculosis detection [5]. In 2021, Creswell and colleagues conducted a study that tested the five certified software programs (CAD4TB (v6), InferRead^®^DR (v2), Lunit INSIGHT CXR (v4.9.0), JF CXR-1 (v2), and qXR (v3)) with cohorts in Bangladesh and found that AI-based software significantly outperformed radiologists in TB detection [6]. However, poor reporting and wide variations in design and methodology limit the reliable interpretation of reported diagnostic accuracy [7]. Furthermore, systematic reviews [8,9] of the diagnostic accuracy of this software also identified several limitations in the available evidence, and uncertainty remains regarding its performance in PTB diagnosis.

Hence, we conducted a systematic review and meta-analysis to synthesize evidence of the accuracy of AI-based software in medical imaging for PTB and to provide new insights for future research.

## 2. Materials and Methods

### 2.1. Data Source and Search Strategy

A MEDLINE and Embase search through the OVID platform was performed on update to November 2022 without any restriction of country. The search terms were built as follows: ‘artificial intelligence’ (deep learning, machine learning, computer assisted, or cnn), ‘imaging’ (radiography, computed tomography, CT, photograph, or X-ray), ‘diagnostic accuracy metrics’ (sensitivity or specificity), and ‘pulmonary tuberculosis’ (Tuberculosis or tb). The full search strategy is laid out in Supplementary Materials File S2. This systematic review was registered in PROSPERO with the number CRD42022379114 and followed the preferred reporting items for systematic reviews and meta-analyses (PRISMA) guidelines (Supplementary Materials File S3).

### 2.2. Study Selection

Two researchers independently assessed the candidate studies for inclusion via screening of titles and abstracts, followed by the full text. Any discrepancy between the two researchers was resolved by a third researcher to achieve a consensus. We included all published studies that used AI-based software to analyze medical imaging in PTB diagnosis. Studies that met the following criteria were included in the final group: (1) Any study that analyzed medical imaging for PTB diagnosis with AI-based software; (2) Studies that provided raw diagnostic accuracy data, sensitivity, specificity, area under curve (AUC), accuracy, negative predictive values (NPVs), or positive predictive values (PPVs). Studies were excluded when they met the following criteria: (1) Case reports, conference reports, reviews, meta-analyses, abstracts without full articles, commentaries/editorials, mathematical modeling studies, and economic analyses; (2) Studies that investigated the accuracy of image segmentation or disease prediction; (3) Triage studies; (4) Studies without outcomes or separate data; (5) Studies that failed to report the source of the included population.

### 2.3. Data Extraction

Two researchers independently extracted demographic and diagnostic-accuracy data using a standardized extraction form from the included studies. When disagreements could not be resolved, we consulted with a third researcher. We extracted data that included study characteristics (first author name, country, year, study design, patient selection methods), demographic information (gender, age, human immunodeficiency virus (HIV) status, drug resistance, history of TB, treatment, imaging modality), AI-based software descriptions (type of artificial intelligence, model, data set, validation methods, threshold score), reference standards, and diagnostic accuracy measures (true and false positives and negatives (TP, FP, FN, TN), AUC, accuracy, sensitivity, specificity, PPV, NPV, and other reported metrics). If there were more than one reported accuracy data set for the same software, with other conditions consistent except for the threshold, the data set with the highest summed sensitivity and specificity would be extracted.

### 2.4. Quality Assessment

The risk of bias and applicability concerns of the included studies were assessed by two researchers separately, with a revised tool developed for diagnostic studies: QUADAS-2. Any disagreement between the two researchers was resolved through discussion with a third researcher.

### 2.5. Data Synthesis and Analysis

Data from development studies and clinical studies were analyzed separately. We first obtained the accuracy data that corresponded to TP, FP, FN, and TN in each included study and calculated the estimated pooled sensitivity, specificity, and AUC associated with the 95% CI, using bivariate random-effects models. Additionally, forest plots of sensitivity and specificity were generated for each study. We also used the model to create a summary receiver operating characteristic (SROC) curve. The I^2^ index was used to assess the heterogeneity between the studies. Values greater than 50% were indicative of substantial heterogeneity [10]. We subsequently chose different study designs, software, reference standards, and AI types as potential sources of heterogeneity, using subgroup analyses to explore the results. A sensitivity analysis was also performed to assess the robustness of the results and identify possible sources of heterogeneity. According to the PRISMA-DTA statement, neither a systematic review nor a meta-analysis of diagnostic accuracy studies is required to assess publication bias [11]. Analyses were conducted in Review Manager version 5.7 and Stata version 17.0 (Stata Corp., College Station, TX, USA), with the midas and metaninf command packages.

## 3. Results

### 3.1. Identification of Studies and Study Characteristics

**Figure 1:**
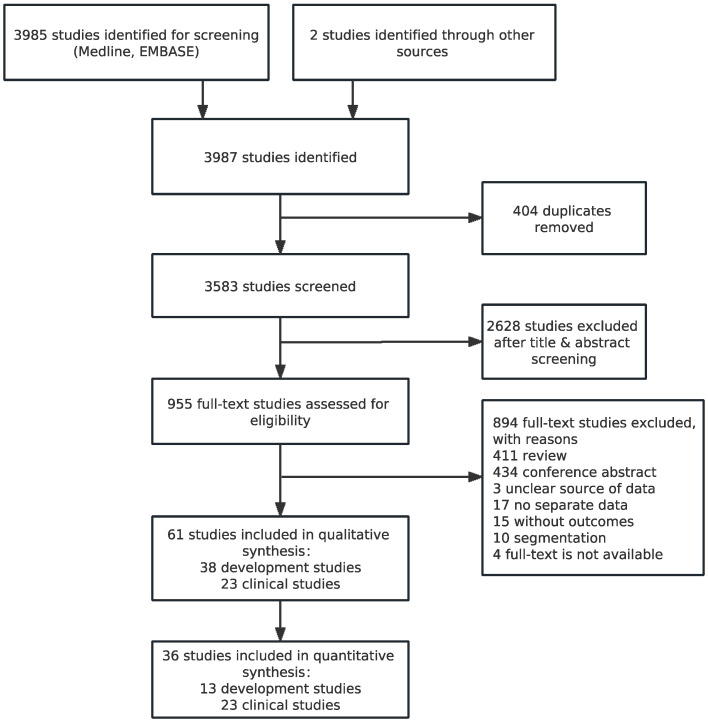
Study flow diagram. Computer-aided detection (CAD).

A total of 3987 articles were identified, of which 404 duplicates and 2628 articles were excluded based on screening of titles and abstracts initially. We then excluded 894 studies upon viewing the full-text articles. Finally, 61 studies (23 clinical and 38 development studies) were included in our descriptive analysis (Table 1) [6,12,13,14,15,16,17,18,19,20,21,22,23,24,25,26,27,28,29,30,31,32,33,34,35,36,37,38,39,40,41,42,43,44,45,46,47,48,49,50,51,52,53,54,55,56,57,58,59,60,61,62,63,64,65,66,67,68,69,70,71]. Due to missing information about the raw diagnostic data from the development studies, we only included 13 development studies, with 18 test evaluation results, in the quantitative analysis (Figure 1) [38,42,43,47,51,54,63,64,66,67,68,69,70].

A total of 50 trials, described in 23 clinical studies, were included in the review, with 124,959 people reporting the diagnostic accuracy of the software used for CXR. No study provided prespecified sample-size calculations. In total, twelve studies [6,13,14,19,21,23,25,26,27,28,32,33] used prospectively collected data, and nine studies [6,17,19,20,21,23,25,26,29] used deep-learning-based versions. Additionally, twelve studies [6,12,14,17,18,19,20,25,26,28,29,30] compared software performance with human readers. Reference standards varied greatly; six studies [14,15,24,25,29,31] compared diagnostic performance with human readers, and fourteen studies [6,12,16,17,19,20,21,22,23,26,27,30,32,33] used microbiological references, while three studies [13,18,28] used both. Notably, some studies evaluated the diagnostic accuracy of AI-based software for special populations. Two studies were conducted on diabetic populations [27,28] and one study included only people from prison [14]. Only fourteen studies [6,13,16,17,20,21,22,23,26,27,28,31,32,33] included their own study populations, and the rest were collected from other studies.

Within the model-development studies, thirty reported diagnostic accuracy for PTB identification with deep-learning-based algorithms, compared with eight studies [34,35,43,50,51,52,53,67] that used machine-learning models. Altogether, twenty-seven out of thirty-eight of the available studies were based on public data sets. Several data sets (Montgomery (NIH), Shenzhen (NIH), and Belarus) were analyzed in most studies, but dataset demographic details were not described in most of the studies. Only one article explicitly described the use of semiautomatic lesion delineation for training data. To validate model performance, nine studies [44,46,48,49,59,60,61,68,70] validated algorithms for external data, while the remaining only implemented internal validation. Considering the economics of practical use, thirty-two out of the thirty-eight studies used CXRs as a diagnostic tool, with CT remaining to be further developed. In addition, eleven studies [36,39,40,41,42,48,52,53,57,60,70] made all of the code used in their implementation freely available to the public. As an important step in the radiomic pipeline, feature extraction played a decisive role in the whole process. Hogeweg, L. et al. [53] combined the results of shape analysis, texture analysis, and focal lesion detection into one combined TB score.

### 3.2. Quality Assessment of Studies

**Figure 2:**
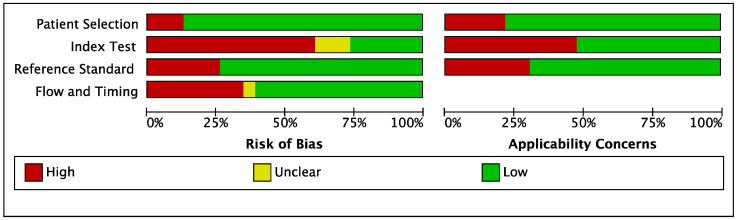
Quality assessment (QUADAS) graph of clinical studies.

**Figure 3:**
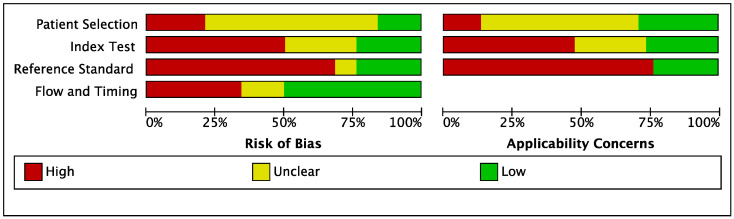
Quality assessment (QUADAS) graph of development studies.

The overall results of the methodological-quality assessment of the included clinical and development studies are summarized respectively in Figure 2 and Figure 3. For clinical studies, the main sources of bias included index tests, flow, and timing. Most development studies were classified as high-risk, particularly with deficiencies in their methods of patient selection, the reference standards used, and their index tests. 

For the patient-selection domain, a high or unclear risk of bias was observed in 84% (thirty-two out of thirty-eight) of the development studies, which was mainly related to missing information in the CXR/CT databases. For the index test, a prespecified threshold was reported only in 30% (seven out of twenty-three) of the clinical studies, and 18% (seven out of thirty-eight) of the development studies had a prespecified threshold, while the other studies had a high risk of bias, since the threshold was determined after the analysis in each. For the reference standard domain, a high or unclear risk of bias was seen in 76% (twenty-nine out of thirty-eight) of the development studies, with regards to assessment by radiologists as the reference standard. For flow and timing, there was a high or unclear risk of bias in 39% (nine out of twenty-three) of the clinical studies and 50% (nineteen out of thirty-eight) of the development studies due to the inconsistency of the reference standards and a lack of inclusion of all patients. 

### 3.3. Diagnostic Accuracy Reported in AI-Based Software Assay for PTB

**Figure 4:**
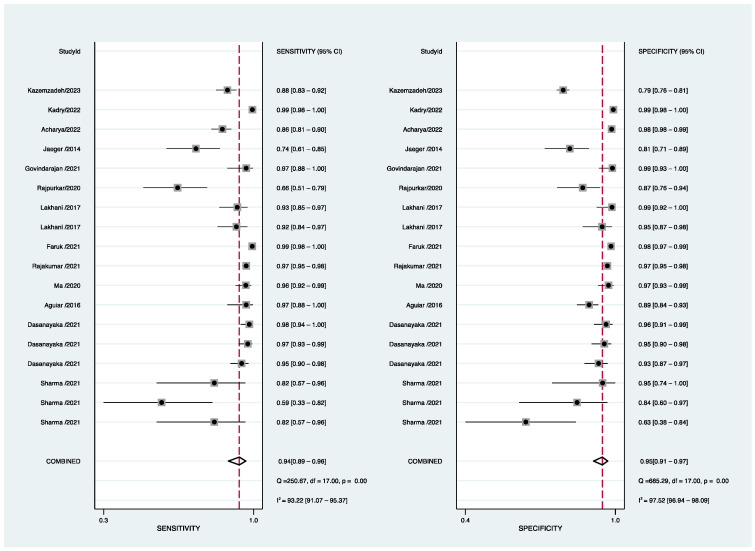
Forest plot of development-study sensitivity and specificity for PTB [38,42,43,47,51,54,63,64,66,67,68,69,70].

We found that only 13 development studies reported TP, FP, FN, and TN for index tests. Of all the 38 articles that included accuracy assessments, the sensitivity ranged from 0.580 to 0.993 and the specificity from 0.570 to 0.996. It is worth noting that CT showed a higher sensitivity in diagnosis with AI (0.750–0.993 of CT vs. 0.580–0.993 of CXR). The reported performance is summarized in Figure 4. The pooled sensitivity of all included studies was 94% (95% CI 89–96%), with I^2^ = 93.22 (95% CI 91.07–95.37), and the pooled specificity was 95% (95% CI 91–97%), with I^2^ = 97.52 (95% CI 96.94–98.09). After excluding the CT-based study, we obtained pooled sensitivity and specificity values of 93% (95% CI 87–96%) and 94% (95% CI 90–97%), respectively.

**Figure 5:**
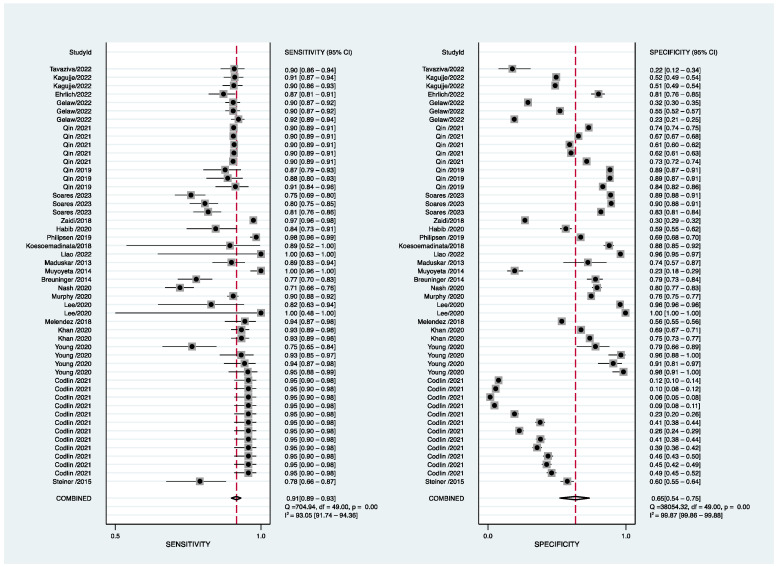
Forest plot of clinical-study sensitivity and specificity for PTB [6,12,13,14,15,16,17,18,19,20,21,22,23,24,25,26,27,28,29,30,31,32,33].

In total, 23 clinical studies, including 124,959 patients, evaluated the diagnostic efficacy of AI programs for PTB. The sensitivity ranged from 0.487 to 1.00, and the pooled sensitivity was 91% (95% CI 89–93%), with I^2^ = 93.05 (95% CI 91.74–94.36). The specificity ranged from 0.063 to 0.997, and the pooled specificity was 65% (95% CI 54–75%), with I^2^ = 99.87 (95% CI 99.86–99.88) (Figure 5). 

There was significant heterogeneity in both sensitivity and specificity. We also constructed SROC curves and calculated the AUC for the included studies. The overall diagnostic performance of the clinical studies and the development studies was comparable [AUC 0.91 (95% CI 0.89–0.94) and 0.98 (95% CI 0.97–0.99), respectively] (Supplementary Materials File S1).

### 3.4. Subgroup and Sensitivity Analyses

Considering the variability of the methods and models tested in the development studies, we only performed a subgroup analysis in the clinical studies, based on predefined parameters, including study design, software, reference standard, and AI type. Some studies were excluded from the relevant subgroup analyses due to missing information or not being categorized into specific groups.

Compared to different study designs, the pooled specificity was 48% (95% CI 34–62%, I^2^ = 99.87; 99.86–99.88) in the prospective assay versus 75% (95% CI 53–89%, I^2^ = 99.94; 99.93–99.94) in the nonprospective assay. When Xpert MTB/RIF was used as the reference standard, the pooled specificity of the Xpert MTB/RIF assay [36% (95% CI 24–50%, I^2^ = 99.93; 99.93–99.94)] was much lower than that of the studies that used human readers [90% (95% CI 80–95%, I^2^ = 98.70; 98.32–99.08)]. Furthermore, the sensitivity and the specificity of various AI-based software (CAD4TB, qXR, Lunit INSIGHT CXR) evidently differed. The results of the subgroup analyses are summarized in detail in Table 2. There was still a substantial level of heterogeneity among each subgroup analysis.

We subsequently performed sensitivity analyses on the clinical and development studies, respectively. Results of our sensitivity analyses are provided in Supplementary Materials File S1. In the clinical studies, we found three articles that had great effects on the overall results. After removal of the corresponding articles, we obtained a still-high heterogeneity (I^2^ = 92.97, 91.55–94.39 for sensitivity, I^2^ = 99.83, 99.82–99.84 for specificity). 

## 4. Discussion

This study sought to (1) evaluate the diagnostic efficacy of AI-based software for PTB and (2) describe the study characteristics, and evaluate the study methodology and the quality of reporting of AI-based software for PTB diagnosis, as well as providing some advice for future software development and clinical applications. Meta-analysis demonstrated that AI-based software has high accuracy in both clinical applications and development studies, indicating that it can assist the physicians in improving the accuracy of PTB diagnosis. However, due to the high heterogeneity and variability between studies, relevant results must be treated with caution when the result of AI-based software is used as a reference standard.

In this systematic review and meta-analysis, we included 23 clinical studies and 38 development studies of PTB diagnosis. Since some missing data were reported, the final count was 13 development studies and 23 clinical studies eligible for quantitative synthesis. Our results show that AI-based software has an excellent ability to diagnose PTB in medical imaging, with pooled sensitivities greater than 0.9 [clinical studies: 91% (95% CI 89%–93%); development studies: 94% (95% CI 89%–96%)]. Additionally, the pooled specificity of the software in the clinical studies was only modest [65% (95% CI 54%–75%)], while that in the development studies was relatively high [95% (95% CI 91%–97%)], which may have been caused by the application of the same test-data set for diagnostic performance assessment. However, a high level of heterogeneity was observed in all the results. Subgroup analysis revealed that nonprospective studies had significantly higher specificity and lower sensitivity than prospective studies had, which might have been due to the inclusion of identified PTB patients in the nonprospective studies. Additionally, studies that used Xpert MTB/RIF as a reference standard had much lower specificity compared to studies that used human readers, possibly because human readers were weaker than Xpert MTB/RIF in correctly identifying negative patients. Furthermore, all commercially available software (CAD4TB, Lunit INSIGHT CXR, and qXR) showed its advantages in improvement of diagnostic accuracy, but we found evident differences in sensitivity and specificity among various AI-based software. The level of heterogeneity between the subgroups remained high, suggesting that study design, software type, AI type, and different reference standards might not be source of heterogeneity. Our follow-up sensitivity analysis indicated that different types of medical imaging might be the sources of heterogeneity, as CT could offer enhanced sensitivity [72].

A number of methodological limitations in the existing evidence were identified, as were study-level factors associated with the reported accuracy, which should all be taken into consideration.

In development studies, most of the current AI-based software was developed for CXR, and only six studies were applied to CT. Because of the deficiency of accuracy data, we performed no subgroup analysis for CT versus CXR. In addition, specific accuracy results, threshold establishment, and inclusion criteria may not have been described well enough to allow emulation for further comparison and may cause greater clinical and methodological heterogeneity. A large proportion of the articles used human readers as the reference standard, meaning systematic overestimation of the diagnostic accuracy of the software. Furthermore, the lack of external validation made it very difficult to formally evaluate algorithm performance. Although most of the experiments used publicly available data sets for model training, few experiments fully disclosed their model details and codes. In addition, almost all of the development articles used manual-lesion-depiction data sets. Semiautomated approaches are known to have greater advantages in lesion delineation, as has been demonstrated with other lung diseases [73], so we encourage more studies in the future to adopt this approach. Several aspects mentioned above lead to the inability to guarantee reproducibility of these experiments. Much of the existing work focuses on multiparametric classification models, ignoring the influence of individual features. Accumulating evidence has confirmed the important role of individual features in discrimination of benign and malignant lung lesions [74,75]; this has great potential for improvement of accuracy and disease identification, and is also informative for research of automated classification models for PTB.

All of the clinical studies evaluated commercially available software developed for CXR. A total of 11 software types were tested, but the version and threshold reported varied among studies. There were varying methodologies of threshold determination and population inclusion, potentially resulting in a high level of heterogeneity. It is worth noting that 13 articles also compared the diagnostic accuracy of AI-based software with human clinicians, which would provide a more objective criterion allowing for a better comparison of models between studies.

Our study had several limitations. Although we searched the relevant literature as comprehensively as possible, some of the literature might have been missed. In addition, some studies failed to report demographic information in detail, and the corresponding subgroup analysis could not be performed. Furthermore, the limited number of studies included for different versions of the software allowed for no further analysis. When AI-based software was used to diagnose PTB, there was significant heterogeneity among studies, so it is difficult to determine whether the software is clinically applicable. Lastly, because current clinical software requires the inclusion of patients over 15 years of age, the diagnostic efficiency for children needs to be further determined.

To improve the future clinical applicability of AI-based software, we recommend that studies include detailed reporting of demographic information, and hope that existing reporting guidelines for diagnostic accuracy studies (STARD) [76] and prediction models (TRIPOD) [77] can be improved as soon as possible to conduct AI-specific amendments. In addition, some model training and validations were performed on CXRs from data sets or sites, potentially resulting in an overestimation of diagnosis power. As such, we suggest that different data sets should be used for model training and testing. Moreover, research teams can collaborate with multiple clinical centers for clinical trials and external validation to make results superior and investigate the stability and heterogeneity of their performance in clinical scenarios. What is more, we appealed to a large number of open, multi-source, and anonymous databases, along with detailed reporting of all of the information needed, such as reference standard, age, HIV status, etc., to fulfill the need for an adequate amount of data with high quality. At the same time, we recommend that development studies make their model details and all of the code used for their experiments freely available to the public to make it possible to reproduce these studies. It is also noteworthy that the diagnostic accuracy of AI-based software should be evaluated against a microbiological reference standard. Lastly, we found a lack of use of AI-based software in CT, and more studies may be needed to explore its superiority in early diagnosis of PTB. In addition, the influence of parameters such as intensity quantization, on imaging and final diagnosis in particular, could be considered.

## 5. Conclusions

In summary, there were relatively high pooled sensitivity and specificity values of AI-based software, which indicates that AI-based software has potential to facilitate diagnosis of PTB in medical imaging, especially in large-scale screening. Heterogeneity was significantly high and extensive variation in reporting, design, and methodology was observed. Thus, standardized reporting guidance around AI-specific trials and multicenter clinical trials is urgently needed to further confirm their stability and heterogeneity in various populations and settings. In the future, we expect more AI-based software with high accuracy to be comprehensively applied for early clinical detection of PTB.

## Figures and Tables

**Table 1:** Methods of studies included in descriptive analysis.

First Author, Year	Imaging Modality	Computer Software/Model	Reference Standard	Accuracy Measures
Maduskar et al., 2013 [12]	CXR	CAD4TB (v 1.08)	AFB smear, MTB culture	TP, FP, TN, FN, AUC, ACC, Sn, Sp, PPV, NPV
Muyoyeta et al., 2014 [13]	CXR	CAD4TB (v 1.08)	Xpert MTB/RIF, human reader	TP, FP, TN, FN, AUC, ACC, Sn, Sp, PPV, NPV
Steiner et al., 2015 [14]	CXR	CAD4TB (v 3.07)	Human reader	AUC
Melendez et al., 2018 [15]	CXR	CAD4TB (v 5)	Human reader	TP, FP, TN, FN, AUC, ACC, Sn, Sp, PPV, NPV
Zaidi et al., 2018 [16]	CXR	CAD4TB (v 3.07)	Xpert MTB/RIF	TP, FP, TN, FN, AUC, ACC, Sn, Sp, PPV, NPV
Qin et al., 2019 [17]	CXR	CAD4TB (v 6), qXR (v 2), Lunit INSIGHT CXR (v 4.7.2)	Xpert MTB/RIF	TP, FP, TN, FN, AUC, ACC, Sn, Sp
Philipsen et al., 2019 [18]	CXR	CAD4TB (v 5)	Xpert MTB/RIF, human reader	TP, FP, TN, FN, AUC, ACC, Sn, Sp, PPV, NPV
Murphy et al., 2020 [19]	CXR	CAD4TB (v 6)	Xpert MTB/RIF	TP, FP, TN, FN, AUC, Sn, Sp
Nash et al., 2020 [20]	CXR	qXR (v 2)	AFB smear, Xpert MTB/RIF or MTB culture	AUC, Sn, Sp
Soares et al., 2023 [21]	CXR	CAD4TB (v 6), Lunit INSIGHT CXR (v 3.1.0.0), qXR (v 3)	Xpert MTB/RIF, MTB culture	AUC, Sn, Sp, PPV, NPV
Qin et al., 2021 [6]	CXR	CAD4TB (v 7), InferRead DR (v 2), Lunit INSIGHT CXR (v 4.9.0), JF CXR-1 (v 2), qXR, (v 3)	Xpert MTB/RIF	AUC, Sn, Sp
Breuninger et al., 2014 [22]	CXR	CAD4TB (v 3.07)	AFB smear, MTB culture	Sn, Sp, PPV, NPV
Khan et al., 2020 [23]	CXR	qXR (v 2), CAD4TB (v 6)	MTB culture	ACC, Sn, Sp, PPV, NPV
Young et al., 2020 [24]	CXR	Not named	Human reader	AUC, Sn, Sp
Liao et al., 2022 [25]	CXR	JF CXR-1 (v 2)	Human reader	TP, FP, TN, FN, AUC, ACC, Sn, Sp, PPV, NPV
Codlin et al., 2021 [26]	CXR	qXR (v 3), CAD4TB (v 7), Genki (v 2), Lunit INSIGHT CXR (v 3.1.0.0), JF CXR-1 (v 3.0), InferRead DR Chest (v 1.0.0.0), ChestEye (v 1), T-Xnet (v 1), XrayAME (v 1), COTO (v 1), SemanticMD (v 1), Dr CADx (v 0.1)	Xpert MTB/RIF	TP, FP, TN, FN, AUC, ACC, Sn, Sp, PPV, NPV
Habib et al., 2020 [27]	CXR	CAD4TB (v 3.07)	Xpert MTB/RIF	AUC, Sn, Sp, PPV, NPV
Koesoemadinata et al., 2018 [28]	CXR	CAD4TB (v 5)	Composite reference standard(s)	AUC, Sn, Sp
Lee et al., 2020 [29]	CXR	Lunit INSIGHT CXR (v 4.7.2)	MTB culture, AFB smear, TB polymerase chain reaction, human reader	TP, FP, TN, FN, AUC, ACC, Sn, Sp, PPV, NPV
Gelaw et al., 2022 [30]	CXR	CAD4TB (v 6), Lunit INSIGHT CXR (v 4.9.0), qXR (v 2)	Xpert MTB/RIF, Mycobacterium tuberculosis (MTB) culture	TP, FP, TN, FN, Sn, Sp
Ehrlich et al., 2022 [31]	CXR	CAD4TB (v 7)	Human reader	TP, FP, TN, FN, AUC, Sn, Sp
Kagujje et al., 2022 [32]	CXR	CAD4TB (v 7), qXR (v 3)	Xpert MTB/RIF	TP, FP, TN, FN, AUC, Sn, Sp
Tavaziva et al., 2022 [33]	CXR	Lunit INSIGHT CXR (v 4.9.0)	Xpert MTB/RIF, Mycobacterium tuberculosis (MTB) culture	TP, FP, TN, FN, AUC, ACC, Sn, Sp
Shen et al., 2010 [34]	CXR	Bayesian classifier	Human reader	ACC
Melendez et al., 2015 [35]	CXR	si-miSVM+PEDD	Human reader	AUC
Pasa et al., 2019 [36]	CXR	CNN	Human reader	AUC, ACC
Xie et al., 2020 [37]	CXR	RCNN	Human reader	AUC, ACC, Sn, Sp
Ma et al., 2020 [38]	CT	U-Net	Sputum smear	AUC, ACC, Sn, Sp, PPV, NPV
Rajpurkar et al., 2020 [39]	CXR	DenseNet	Xpert MTB/RIF, MTB culture	ACC, Sn, Sp
Oloko-Oba et al., 2021 [40]	CXR	EfficientNets	Human reader	AUC, ACC, Sn, Sp
Mamalakis et al., 2021 [41]	CXR	DenseNet-121, ResNet-50	Human reader	AUC, F1, precision, recall
Rajakumar et al., 2021 [42]	CXR	VGG16, VGG19, KNN	Human reader	ACC, Sn, Sp, NPV
Sharma et al., 2021 [43]	CXR	Tree, SVM, Naïve Bayes	Composite reference standard(s)	AUC, F1, CA, precision, recall
Wang et al., 2021 [44]	CT	3D-ResNet	AFB smear, MTB culture	AUC, Sn, Sp, ACC, F1
Showkatian et al., 2022 [45]	CXR	ConvNet	Human reader	AUC, ACC, F1, precision, recall
Zhou et al., 2022 [46]	CXR	ResNet	Human reader	AUC, ACC, Sn, Sp, PPV, NPV
Rajaraman et al., 2021 [47]	CXR	ImageNet, VGG-16	Human reader	AUC, ACC, Sn, Sp, F1, precision
Yan et al., 2021 [48]	CT	SeNet-ResNet-18	Human reader	ACC, precision, recall
Zhang et al., 2021 [49]	CT	CBIR-CSNN	Composite reference standard(s)	AUC, ACC
Arzhaeva et al., 2009 [50]	CXR	MVDB	Human reader	AUC
Jaeger et al., 2014 [51]	CXR	SVM	Human reader	AUC, ACC
Chauhan et al., 2014 [52]	CXR	SVM	Human reader	AUC, ACC, Sn, Sp, F1, precision
Hogeweg et al., 2015 [53]	CXR	RF50, GB50, LDA, KNN13	MTB culture, human reader	AUC
Lakhani et al., 2017 [54]	CXR	AlexNet, GoogLeNet	Human reader	AUC, ACC, Sn, Sp
Han et al., 2021 [55]	CXR	VGG16	Human reader	AUC, Sn, Sp
An et al., 2022 [56]	CXR	E-TBNet (ResNet)	Human reader	ACC, Sn, Sp, NPV, ppv, F1
Lee et al., 2021 [57]	CXR	EfficientNet	Xpert MTB/RIF, MTB culture, human reader	AUC
Khatibi et al., 2021 [58]	CXR	CNN, CCNSE	Human reader	AUC, ACC
Kim et al., 2020 [59]	CXR	DCNN	Human reader	AUC, Sn, Sp, NPV, PPV, F1
Feng et al., 2020 [60]	CT	CNN	Composite reference standard(s)	AUC, ACC, Sn, Sp
Hwang et al., 2019 [61]	CXR	CNN	Human reader	AUC, Sn, Sp
Heo et al., 2019 [62]	CXR	I-CNN(VGG19), D-CNN(VGG19)	Human reader	AUC
Aguiar et al., 2016 [63]	CXR	MLP	Human reader	AUC, Sn, Sp, PPV, NPV
Faruk et al., 2021 [64]	CT	Xception, InceptionV3, InceptionResNetV2, MobileNetV2	Human reader	Sn, precision, recall, F1
Karki et al., 2021 [65]	CXR	InceptionV3, Xception	Human reader	AUC
Dasanayaka et al., 2021 [66]	CXR	VGG16, InceptionV3, Ensemble	Human reader	ACC, Sn, Sp
Govindarajan et al., 2021 [67]	CXR	ELM, OSELM	Human reader	Sn, Sp, precision, F1
Acharya et al., 2022 [68]	CXR	ImageNet fine-tuned normalization-free networks	Human reader	Sn, Sp, AUC, ACC, precision, recall
Kadry et al., 2022 [69]	CXR	VGG16, Fine Tree	Xpert MTB/RIF, Mycobacterium tuberculosis (MTB) culture, human reader	Sn, Sp, ACC, NPV
Kazemzadeh et al., 2023 [70]	CXR	NR	Human reader	Sn, Sp, AUC
Margarat et al., 2022 [71]	CXR	DBN-AMBO	Human reader	Sp, ACC, precision, recall, NPV

Abbreviations: CXR, chest X-ray; CT, computed tomography; CAD, computer-aided detection; CNN, convolutional neural networks; RCNN, regions with CNN features; KNN, K-nearest neighbor; VGG, visual geometry group; SVM, support vector machine; HIV, human immunodeficiency virus; DLAD, deep-learning-based automatic detection; AFB, acid-fast bacilli; MTB, Mycobacterium tuberculosis; TP, true positive; FP, false positive; TN, true negative; FN, false negative; AUC, area under the receiver operating curve; ACC, accuracy; Sn, sensitivity; Sp, specificity; CA, cluster accuracy; DBN-AMBO, deep belief network with adaptive monarch butterfly optimization.

**Table 2:** Subgroup analysis based on different standards.

Studies	Sensitivity (95%CI)	Specificity (95%CI)	DOR (95%CI)	AUC (95%CI)
All (23)	0.91(0.89–0.93)	0.65(0.55–0.75)	20(13–29)	0.91(0.89–0.94)
Study Design				
Prospective (12)	0.91(0.87–0.94)	0.48(0.34–0.62)	9(4–20)	0.85(0.82–0.88)
Nonprospective (11)	0.87(0.78–0.93)	0.75(0.53–0.89)	20(5–84)	0.90(0.87–0.92)
Software				
CAD4TB (18)	0.89(0.82–0.94)	0.57(0.42–0.70)	11(4–30)	0.83(0.80–0.86)
qXR (8)	0.79(0.61–0.90)	0.55(0.24–0.83)	5(1–38)	0.77(0.73–0.80)
Lunit INSIGHT CXR (8)	0.88(0.75–0.94)	0.78(0.40–0.95)	25(3–211)	0.91(0.88–0.93)
Reference standard				
Human reader (5)	0.90(0.84–0.94)	0.90(0.80–0.95)	77(22–269)	0.95(0.93–0.97)
Xpert MTB/RIF (9)	0.90(0.85–0.93)	0.36(0.24–0.50)	5(2–12)	0.79(0.75–0.82)
AI type				
Deep learning (13)	0.91(0.89–0.92)	0.62(0.48–0.74)	16(10–23)	0.91(0.88–0.93)
Machine learning (9)	0.93(0.85–0.97)	0.61(0.46–0.75)	21(11–42)	0.87(0.83–0.89)

Abbreviation: DOR, diagnostic odds ratio; AUC, area under curve.

## Data Availability

Not applicable.

## Supplementary Materials

The following supporting information can be downloaded at: https://www.mdpi.com/article/10.3390/jcm12010303/s1, Supplementary Materials File S1: Figure S1 Quality assessment (QUADAS 2) summary of clinical studies: risk of bias & applicability concerns; Figure S2 Quality assessment (QUADAS 2) summary of development studies: Risk of bias and applicability concerns; Figure S3 Sensitivity analysis of clinical studies; Figure S4 Sensitivity analysis of development studies; Figure S5 Summary receiver operating characteristic (SOC) curve of clinical studies; Figure S6 Summary receiver operating characteristic (SROC) curve of development studies; Table S1 Demographics of clinical studies; Table S2 Accuracy information of clinical studies included; Table S3 Accuracy measures reported by development studies; Table S4 Accuracy measures reported by development studies; Supplementary Materials File S2: Search strategy; Supplementary Materials File S3: PRISMA checklist [78].
