# Supplementary material for: Diagnostic Accuracy of the Artificial Intelligence Methods in Medical Imaging for Pulmonary Tuberculosis: A Systematic Review and Meta-Analysis
Source: J Clin Med. 2022 Dec 30;12(1):303. doi: 10.3390/jcm12010303 (PMC9820940; doi:10.3390/jcm12010303)
Supplement: Supplementary file 1 [file jcm-12-00303-s001.zip › Supplementary Materials File S1.pdf]

Supplementary Materials

Supplementary Materials S1

	Risk of Bias				Applicability Concerns		
	Patient Selection	Index Test	Reference Standard	Flow and Timing	Patient Selection	Index Test	Reference Standard
A. Steiner 2015	+	–	–	+	+	–	–
Andrew J. Codlin 2021	+	?	+	+	+	+	+
C. Young 2020	–	–	–	–	–	–	–
Faiz Ahmad Khan 2020	+	?	+	+	+	+	+
Jaime. Melendez 2018	+	–	–	?	–	+	–
Jong Hyuk Lee 2020	+	–	+	+	+	–	+
Keelin Murphy 2020	+	–	+	–	+	–	+
Madlen nash 2020	–	–	+	–	–	–	+
Marianne Breuninger 2014	+	–	+	–	+	+	+
Monde Muyoyeta 2014	–	+	+	–	+	+	+
P . Maduskar 2013	+	+	+	+	+	+	+
Qinghua Liao 2022	+	–	+	+	+	+	+
R. C. Koesoemadinata 2018	+	–	+	+	–	–	+
R. H. H. M. Philipsen 2019	+	+	+	–	+	+	+
Shifa Salman Habib 2020	+	?	+	+	–	+	+
Syed Mohammad Asad Zaidi 2018	+	+	–	–	+	+	–
Thiego Ramon Soares 2023	+	+	+	–	+	+	–
Zhi Zhen Qin 2019	+	–	+	+	+	–	+
Zhi Zhen Qin 2021	+	–	+	+	+	–	+

– High ? Unclear + Low

Figure S1. Quality assessment (QUADAS 2) summary of clinical studies: risk of bias & applicability concerns.

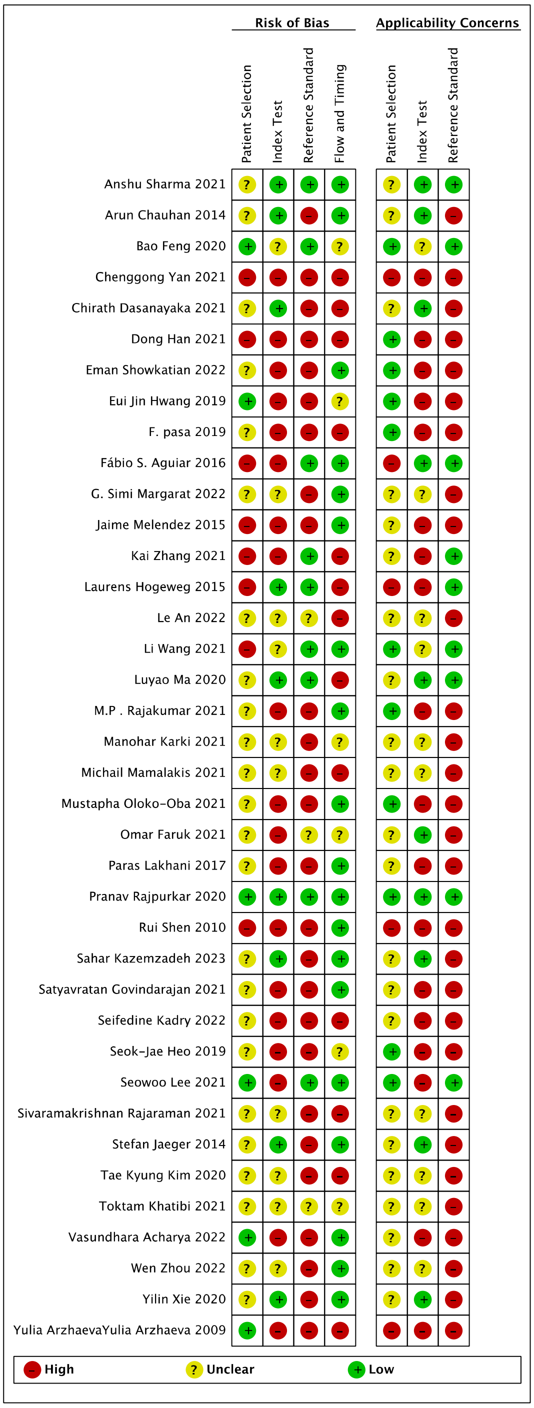

Figure S2. Quality assessment (QUADAS 2) summary of development studies: Risk of bias and applicability concerns.

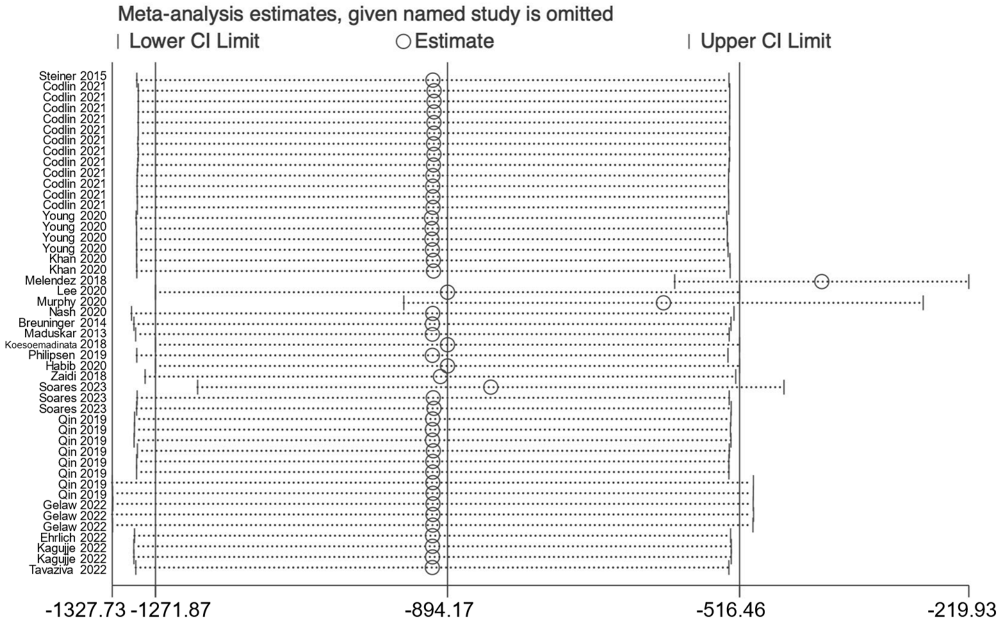

Figure S3. Sensitivity analysis of clinical studies.

*** We excluded three sets of data (Lee 2020, Liao 2022, Muyoyeta 2014) for which sensitivity analysis could not be performed.**

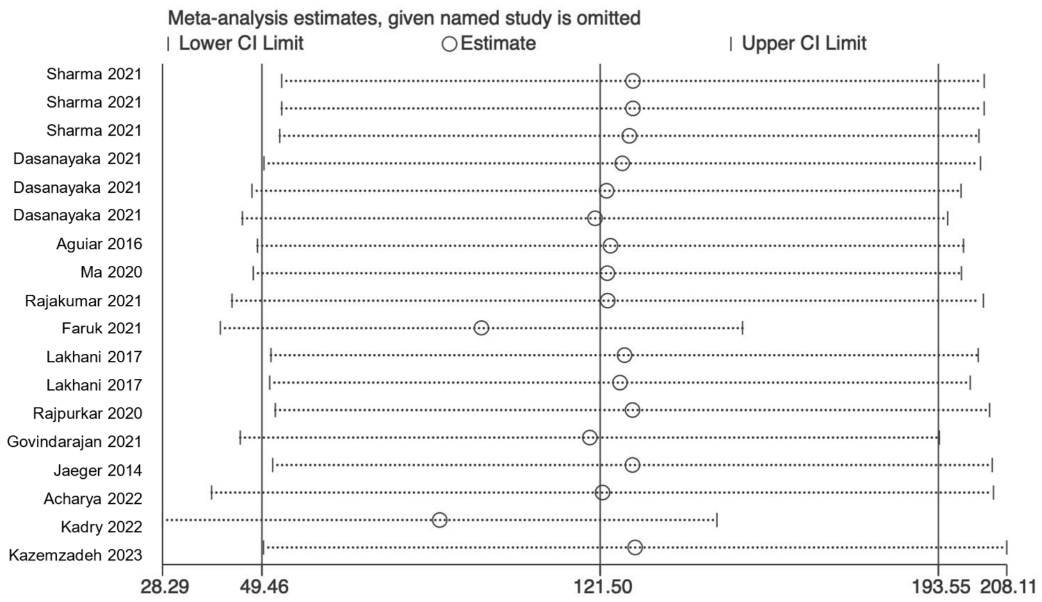

Figure S4. Sensitivity analysis of development studies.

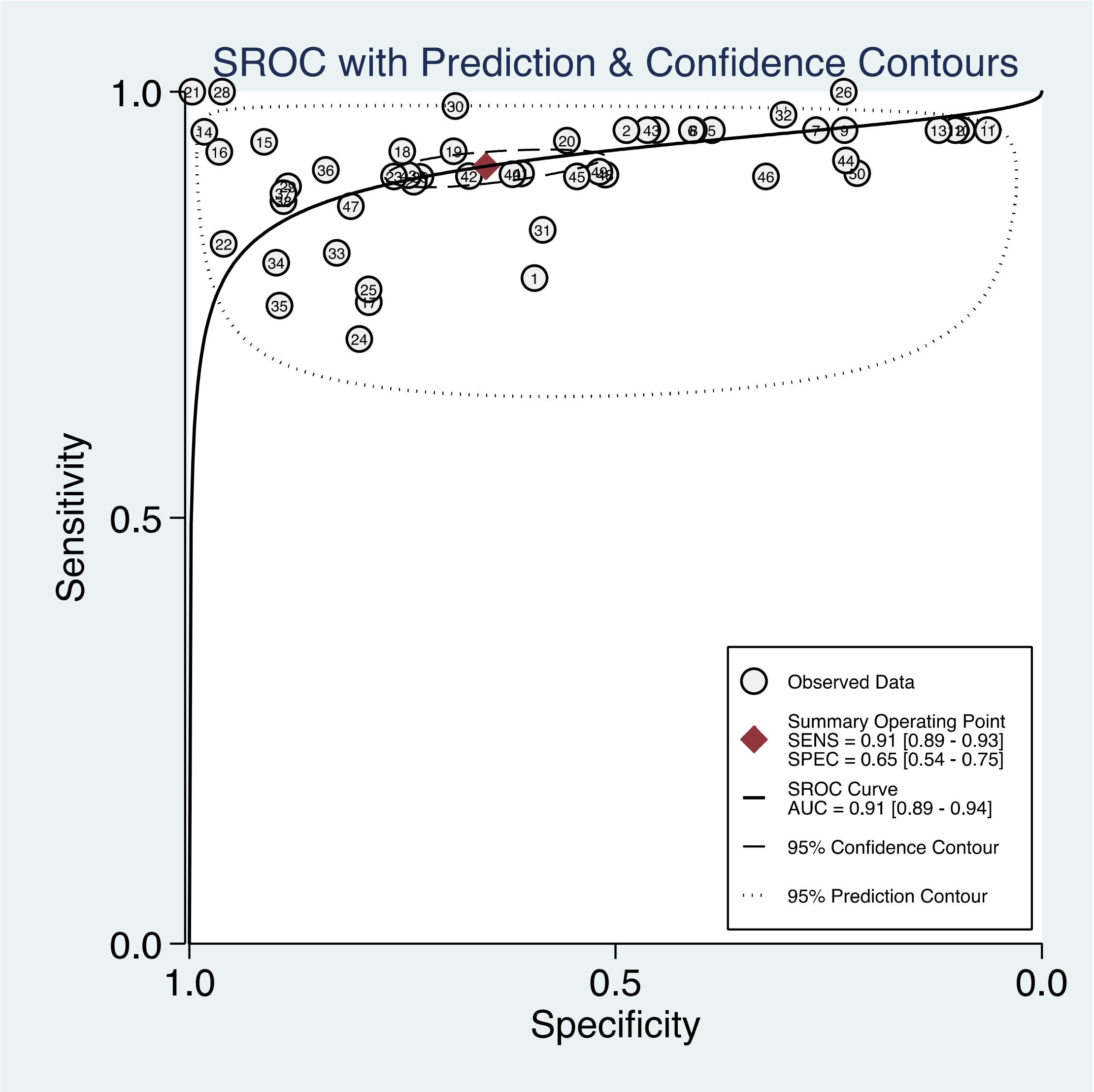

Figure S5. Summary receiver operating characteristic (SROC) curve of clinical studies.

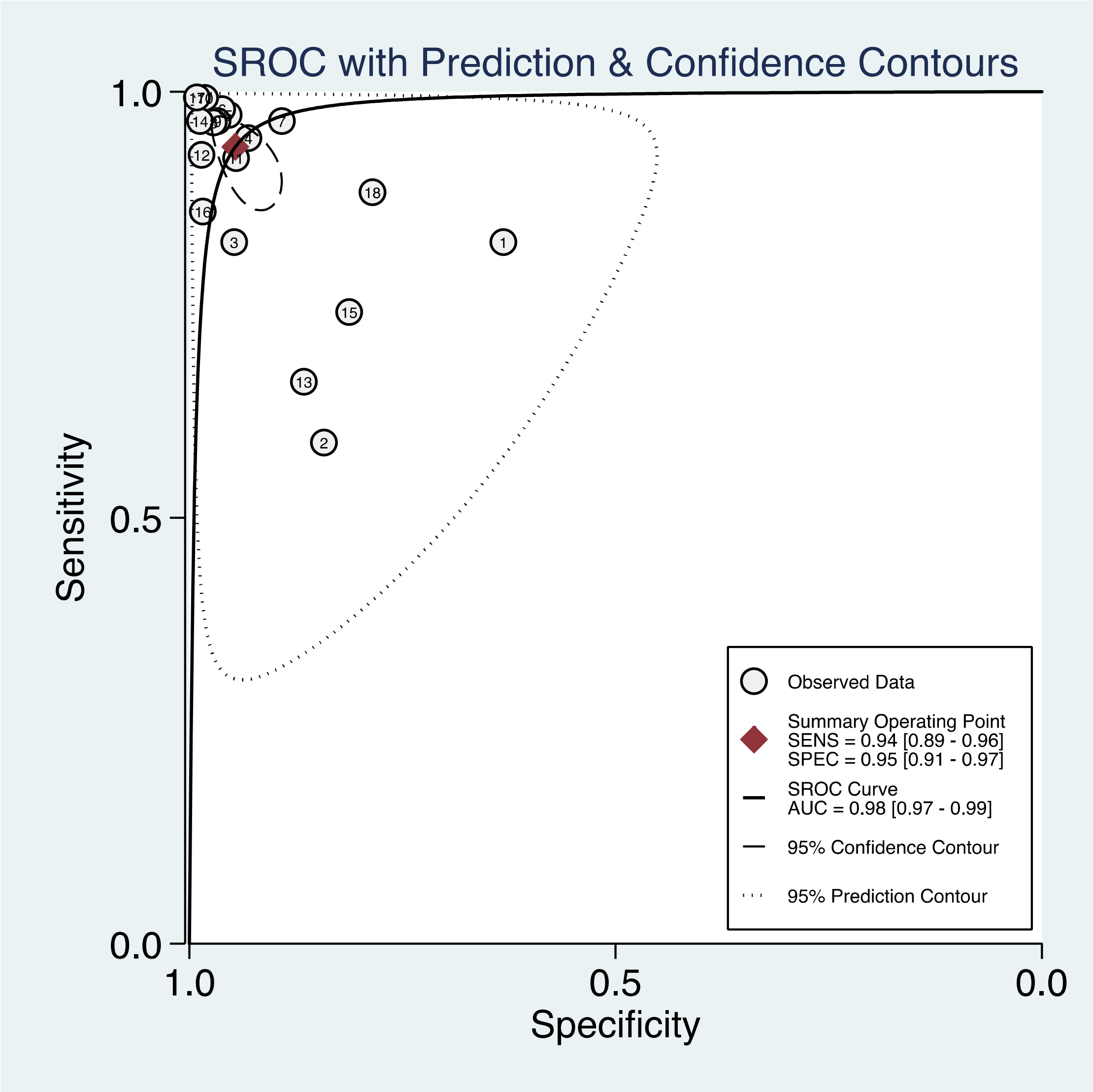

Figure S6. Summary receiver operating characteristic (SROC) curve of development studies.

Table S1. Demographics of clinical studies.

First author, year	Country	All	Sex		Average age	Previous TB		HIV	
			Male (%)	Female (%)		Yes N (%)	No N (%)	Positive N (%)	Negative N (%)
**Deep learning**									
Qin et al, 2019	Nepal, Cameroon	1196	550 (46)	646 (54)	46	NR	NR	38 (3)	271 (23)
Murphy et al, 2020	Pakistan	5565	2756 (50)	2809 (50)	NR	NR	NR	NR	NR
Nash et al, 2020	India	929	647 (70)	282 (30)	51.9	138 (15)	NR	47 (5)	NR
Soares et al, 2023	Brazil	2075	NR	NR	33	293	NR	NR	NR
Qin et al, 2021	Bangladesh	23954	16078 (67)	7876 (33)	42	3586 (15)	NR	NR	NR
Khan et al, 2020	Pakistan	2198	1148 (52)	1050 (48)	NR	499 (23)	NR	NR	2187 (99)
Liao et al, 2022	China	2543	1073 (42)	1470 (58)	NR	8 (0.3)	2535 (99.7)	NR	NR
Codlin et al, 2021	Vietnam	1032	712 (69)	320 (31)	62	NR	NR	2 (0.2)	NR
Lee et al, 2020	South Korea	20135	20135 (100)	0 (0)	NR	NR	NR	NR	NR
Gelaw et al, 2022	Africa, Asia, Middle East, Eastern Europe	1769	1071 (61)	698 (39)	NR	251 (14)	1518 (86)	18 (1)	NR
Ehrlich et al, 2022	South Africa	501	NR	NR	NR	NR	NR	NR	NR
Kagujje et al, 2022	Zambia	1884	1184 (63)	700 (37)	38	452 (24)	1432 (76)	702 (37)	1073 (57)
Tavaziva et al, 2020	Pakistan	2190	1144 (52)	1046 (48)	33	495 (23)	1692 (77)	NR	NR
**Machine learning**									
Maduskar et al, 2013	WHO TB Specimen Bank	161	42 (26)	119 (74)	35.8±9.6	NR	NR	110 (68)	NR
Muyoyeta et al, 2014	Zambia	350	215 (61)	135 (39)	36.5	78 (22)	272 (78)	190 (54)	148 (42)
Steiner et al, 2015	Tanzanian	511	511 (100)	0 (0)	37	NR	NR	58 (11)	402 (79)
Melendez et al, 2018	UK	39328	39328 (100)	0 (0)	NR	NR	NR	NR	NR
Zaidi et al, 2018	Pakistan	6090	3018 (50)	3072 (50)	NR	NR	NR	NR	NR
Philipsen et al, 2019	Philippines	10755	NR	NR	NR	NR	NR	NR	NR
Breuninger et al, 2014	Tanzania	861	428 (50)	433 (50)	42	144 (17)	717 (83)	379 (44)	478 (56)
Habib et al, 2020	Pakistan	694	374 (54)	320 (46)	54	74 (11)	620 (89)	NR	NR
Koesoemadinata et al, 2018	Indonesia	794	291 (37)	503 (63)	NR	82 (10)	712 (90)	2 (0.3)	3 (0.4)
**Not reported**									
Young et al, 2020	MHSC, MBOD	142	142 (100)	0 (0)	NR	NR	NR	NR	NR

Abbreviation: TB, tuberculosis; HIV, human immunodeficiency virus; NR, not reported; WHO, world health organization; UK, United Kingdom; MHSC, mine health and safety council; MBOD, medical bureau for occupational diseases.

Table S2. Accuracy information of clinical studies included.

First author, year	Imaging modality	Software	Threshold	Reference standard	AUC	TP	FP	FN	TN	Sensitivity	Specificity
**Deep learning**											
**Qin et al, 2019**	CXR	CAD4TB (v 6),<br>qXR (v 2),<br>Lunit INSIGHT CXR(v 4.7.2)	63(CAD4TB (v 6)),<br>0.67(qXR(v 2)),<br>0.92(Lunit INSIGHT CXR (v 4.7.2))	Xpert MTB/RIF	0.920(CAD4TB (v 6)),<br>0.940(qXR(v 2)),<br>0.940(Lunit INSIGHT CXR (v 4.7.2))	99<br>96<br>95	174<br>120<br>120	10<br>13<br>14	913<br>967<br>967	0.910(CAD4TB (v 6)),<br>0.880(qXR(v 2)),<br>0.870(Lunit INSIGHT (v 4.7.2))	0.840(CAD4TB (v 6)),<br>0.890(qXR(v 2)),<br>0.890(Lunit INSIGHT CXR (v 4.7.2))
**Murphy et al, 2020**	CXR	CAD4TB (v 6)	NR	Xpert MTB/RIF	0.885	769	1131	85	3580	0.900	0.760
**Nash et al, 2020**	CXR	qXR (v 2)	0.818	AFB smear, Xpert MTB/RIF or MTB culture	0.810	225	122	92	490	0.710	0.800
**Soares et al, 2023**	CXR	CAD4TB (v 6),<br>Lunit INSIGHT CXR (v 3.1.0.0),<br>qXR (v 3)	60(CAD4TB (v 6)),<br>0.72(Lunit INSIGHT CXR (v 3.1.0.0)),<br>0.5(qXR (v 3))	Xpert MTB/RIF, MTB culture	0.877(CAD4TB (v 6)),<br>0.912(Lunit INSIGHT CXR (v 3.1.0.0)),<br>0.901(qXR (v3))	210<br>207<br>194	314<br>185<br>192	49<br>52<br>65	1502<br>1631<br>1624	0.807(CAD4TB (v 6)),<br>0.799(Lunit INSIGHT CXR (v 3.1.0.0)),<br>0.745(qXR (v3))	0.827(CAD4TB (v 6)),<br>0.898(Lunit INSIGHT CXR (v 3.1.0.0)),<br>0.894(qXR (v3))
**Qin et al，2021**	CXR	CAD4TB (v 7),<br>InferRead DR (v 2),<br>Lunit INSIGHT CXR (v 4.9.0),<br>JF CXR-1 (v 2),<br>qXR (v 3)	50(CAD4TB (v 7)),<br>0.34(InferRead DR (v 2)),<br>0.92(Lunit INSIGHT CXR (v 4.9.0)),<br>0.6(JF CXR-1 (v 2)),<br>0.6(qXR (v 3))	Xpert MTB/RIF	0.903(CAD4TB (v 7)),<br>0.849(InferRead DR (v 2)),<br>0.849(Lunit INSIGHT CXR (v 4.9.0)),<br>0.886(JF CXR-1 (v 2)),<br>0.908(qXR (v 3))	3308<br>3319<br>3322<br>3311<br>3315	5496<br>7686<br>7889<br>6652<br>5212	36714783<br>35612593<br>35312390<br>36413627<br>36015067		0.900(CAD4TB (v 7)),<br>0.903(InferRead DR (v 2)),<br>0.904(Lunit INSIGHT CXR (v 4.9.0)),<br>0.901(JF CXR-1 (v 2)),<br>0.902(qXR (v 3))	0.729(CAD4TB (v 7)),<br>0.621(InferRead DR (v 2)),<br>0.611(Lunit INSIGHT CXR (v 4.9.0)),<br>0.672(JF CXR-1 (v 2)),<br>0.743(qXR (v 3))
**Khan et al, 2020**	CXR	qXR (v 2),<br>CAD4TB (v 6)	0.49(qXR (v 2)),<br>NR (CAD4TB (v 6))	MTB culture	NR<br>NR	253<br>253	481<br>597	19<br>19	1445<br>1329	0.930(qXR (v 2)),<br>0.930(CAD4TB (v 6))	0.750(qXR (v 2)),<br>0.690(CAD4TB (v 6))
**Liao et al, 2022**	CXR	JF CXR-1 (v 2)	30	Human reader	0.987	8	97	0	2438	1.000	0.957
**Codlin et al, 2021**	CXR	qXR (v 3),<br>CAD4TB (v 7),<br>Genki (v 2),<br>Lunit INSIGHT CXR (v 3.1.0.0),<br>JF CXR-1 (v 3.0),<br>InferRead DR Chest (v 1.0.0.0),<br>ChestEye (v 1),<br>T-Xnet (v 1),<br>XrayAME (v 1),<br>COTO (v 1),<br>SemanticMD (v 1),<br>Dr CADx (v 0.1)	44.1(qXR (v 3)),<br>31.1(CAD4TB (v 7)),<br>46.7(Genki (v 2)),<br>3(Lunit INSIGHT CXR (v 3.1.0.0)),<br>83.4(JF CXR-1 (v 3.0)),<br>53.8(InferRead DR Chest (v 1.0.0.0)),<br>15.4(ChestEye (v 1)),<br>1.2(T-Xnet (v 1)),<br>0.6(XrayAME (v 1)),<br>1.5(COTO (v 1)),<br>0.4(SemanticMD (v 1)),<br>27.8(Dr CADx (v 0.1))	Xpert MTB/RIF	0.820(qXR (v 3)),<br>0.820(CAD4TB (v 7)),<br>0.780(Genki (v 2)),<br>0.820(Lunit INSIGHT CXR (v 3.1.0.0)),<br>0.770(JF CXR-1 (v 3.0)),<br>0.760(InferRead DR Chest (v 1.0.0.0)),<br>0.730(ChestEye (v 1)),<br>0.700(T-Xnet (v 1)),<br>0.660(XrayAME (v 1)),<br>0.660(COTO (v 1)),<br>0.530(SemanticMD (v 1)),<br>0.500(Dr CADx (v 0.1))	127<br>127<br>127<br>127<br>127<br>127<br>127<br>127<br>127<br>127<br>127<br>127	461<br>492<br>483<br>551<br>530<br>661<br>532<br>691<br>815<br>842<br>808<br>790	6<br>6<br>6<br>6<br>6<br>6<br>6<br>6<br>6<br>6<br>6<br>6	438<br>407<br>416<br>348<br>369<br>238<br>367<br>208<br>84<br>57<br>91<br>109	0.955(qXR (v 3)),<br>0.955(CAD4TB (v 7)),<br>0.955(Genki (v 2)),<br>0.955 (Lunit INSIGHT CXR (v 3.1.0.0)),<br>0.955(JF CXR-1 (v 3.0)),<br>0.955(InferRead DR Chest (v 1.0.0.0)),<br>0.955(ChestEye (v 1)),<br>0.955(T-Xnet (v 1)),<br>0.955(XrayAME (v 1)),<br>0.955(COTO (v 1)),<br>0.955(SemanticMD (v 1)),<br>0.955(Dr CADx (v 0.1))	0.450(qXR (v 3)),<br>0.453(CAD4TB (v 7)),<br>0.463(Genki (v 2)),<br>0.387(Lunit INSIGHT CXR (v 3.1.0.0)),<br>0.410(JF CXR-1 (v 3.0)),<br>0.265(InferRead DR Chest (v1.0.0.0)),<br>0.408(ChestEye (v 1)),<br>0.231(T-Xnet (v 1)),<br>0.093(XrayAME (v 1)),<br>0.063(COTO (v 1)),<br>0.101(SemanticMD (v 1)),<br>0.121(Dr CADx (v 0.1))

**Lee et al, 2020**	CXR	Lunit INSIGHT CXR (v 4.7.2)	0.46, 0.16	MTB culture, AFB smear, TB polymerase chain reaction, human reader	1.000, 0.967	5<br>23	69<br>809	0<br>5	20061<br>19298	1.000, 0.821	0.997, 0.960
**Gelaw et al, 2022**	CXR	CAD4TB (v 6), Lunit INSIGHT CXR (v 4.9.0.0), qXR (v 2)	46(CAD4TB (v 6))<br>0.076(Lunit INSIGHT CXR (v 4.9.0.0))<br>0.29(qXR (v 2))	Xpert MTB/RIF, Mycobacterium tuberculosis (MTB) culture	NR	490<br>480<br>480	952<br>562<br>836	43<br>53<br>53	284<br>674<br>400	0.902 (CAD4TB (v 6))<br>0.901(Lunit INSIGHT CXR (v 4.9.0.0))<br>0.901(qXR (v 2))	0.230(CAD4TB (v 6))<br>0.545(Lunit INSIGHT CXR (v 4.9.0.0))<br>0.324(qXR (v 2))
**Ehrlich et al, 2022**	CXR	CAD4TB (v 7)	NR	Human reader	0.889	187	54	29	231	0.867	0.812
**Kagujje et al, 2022**	CXR	CAD4TB (v 7), qXR (v 3)	17(CAD4TB (v 7))<br>6(qXR (v 3))	Xpert MTB/RIF	0.850(CAD4TB (v 7))<br>0.860(qXR (v 3))	269<br>270	774<br>763	29<br>28	812<br>823	0.903 (CAD4TB (v 7))<br>0.906(qXR (v 3))	0.512(CAD4TB (v 7))<br>0.519(qXR (v 3))
**Tavaziva et al, 2022**	CXR	Lunit INSIGHT CXR (v 3.1.0.0)	30	Xpert MTB/RIF, Mycobacterium tuberculosis (MTB) culture	0.850	189	47	20	13	0.877	0.643
**Machine learning**											
**Maduskar et al, 2013**	CXR	CAD4TB (v 1.08)	50	AFB smear, MTB culture	0.730	110	10	13	28	0.880	0.410
**Muyoyeta et al, 2014**	CXR	CAD4TB (v 1.08)	60	Xpert MTB/RIF, human reader	0.710	96	195	0	59	1.000	0.232
**Steiner et al, 2015**	CXR	CAD4TB (v 3.07)	70	Human reader	0.750	50	181	14	266	0.783	0.594
**Zaidi et al, 2018**	CXR	CAD4TB (v 3.07)	50	Xpert MTB/RIF	0.790	900	3600	25	1565	0.973	0.303
**Breuninger et al, 2014**	CXR	CAD4TB (v 3.07)	74	AFB smear, MTB culture	NR	149	49	45	184	0.770	0.790
**Habib et al, 2020**	CXR	CAD4TB (v 3.07)	60	Xpert MTB/RIF	0.780	62	257	12	363	0.840	0.586
**Melendez et al, 2018**	CXR	CAD4TB (v 5)	39.8	Human reader	0.900	82	17218	5	21656	0.950	0.560
**Philipsen et al, 2019**	CXR	CAD4TB (v 5)	60	Xpert MTB/RIF, human reader	0.930	293	3263	5	7194	0.980	0.690
**Koesoemadinata et al, 2018**	CXR	CAD4TB (v 5)	65	Composite reference standard(s)	0.890	8	39	1	298	0.889	0.884
**Not reported**											
**Young et al, 2020**	CXR	Not named	NR	Human reader	0.989,<br>0.963,<br>0.980,<br>0.768	81<br>80<br>79<br>64	1<br>5<br>2<br>12	4<br>5<br>6<br>21	56<br>52<br>55<br>45	0.953,<br>0.941,<br>0.929,<br>0.753	0.982,<br>0.912,<br>0.965,<br>0.789

Abbreviation: CXR, chest X-ray; CT, computed tomography; AFB, acid fast bacilli; AUC, area under the receiver operating curve; TP, true positive; FP, false positive; FN, false negative; TN, true negative; MTB, Mycobacterium tuberculosis; NR, not reported.

Table S3. Accuracy measures reported by development studies.

First author, year	Model	Imaging modality	Database used	training set	Type of internal validation	External validation（Yes or No）	Test set	Reference standard
Pasa et al, 2019	CNN	CXR	MC, CH, Belarus	1523	5-fold cross-validation	No	381	Human reader
Xie et al, 2020	RCNN	CXR	JSRT, MC, CH, FANXJU	5184	5-fold cross-validation	No	NR	Human reader
Ma et al, 2020	U-Net	CT	Hebei	567	NR	No	279	Sputum smear
Rajpurkar et al, 2020	DenseNet	CXR	Jooste/Khayelitsha	563	5-fold cross-validation	No	114	Xpert MTB/RIF, MTB culture
Oloko-Oba et al, 2021	EfficientNets	CXR	MC, CH	595	NR	No	201	Human reader
Mamalakis et al, 2021	DenseNet-121, ResNet-50	CXR	Pediatric CXRs dataset, IEEE COVID-19 CXRs dataset, CH	1009	Monte Carlo cross validation split	No	303	Human reader
Rajakumar et al, 2021	VGG16, VGG19, KNN	CXR	MC, CH, NLM, RSNA	4000	5-fold cross-validation	No	NR	Human reader
Wang et al, 2021	3D-ResNet	CT	Tianjin, Xi`an	886	Hold-out method	Yes	106	AFB smear, MTB culture
Showkatian et al, 2022	ConvNet	CXR	MC, CH	2040	random split	No	120	Human reader
Zhou et al, 2022	ResNet	CXR	CHNCXR, MCUCXR, China	5045	NR	Yes	1349	Human reader
Rajaraman et al, 2021	ImageNet, VGG-16	CXR	CH, MC	384	4-fold cross-validation	No	NR	Human reader
Yan et al, 2021	SeNet-ResNet-18	CT	NH, YH, HH, NIH	714	Hold-out method	Yes	99/86/171(NH, YH), 86(NH, HH), 171(NH, NIH)	Human reader
Zhang et al, 2021	CBIR-CSNN	CT	Liaoning	2356	Hold-out method	Yes	300	Composite reference standard(s)
Lakhani et al, 2017	AlexNet, GoogLeNet	CXR	MC, CH, Belarus, Thomas Jefferson University Hospital	1370	random split	No	300	Human reader
Han et al, 2021	VGG16	CXR	China	1992	random split	No	796	Human reader
An et al, 2022	E-TBNet (ResNet)	CXR	MC, CH	111	Hold-out method	No	27	Human reader
Lee et al, 2021	EfficientNet	CXR	Korean hospitals	6883	Hold-out method	No	3922	Xpert MTB/RIF, MTB culture, Human reader
Khatibi et al, 2021	CNN, CCNSE	CXR	MC, CH	753	5-fold cross-validation	No	NR	Human reader
Kim et al, 2020	DCNN	CXR	NIH CXR, NIH CXR, MC, SZ, Johns Hopkins Hospital, Baltimore, MD, USA	111622	Hold-out method	Yes	1800	Human reader

Feng et al, 2020	CNN	CT	China	218	random split	Yes	NR	Composite reference standard(s)
Hwang et al, 2019	CNN	CXR	Seoul National University Hospital, Korea	60089	random split	Yes	450	Human reader
Heo et al, 2019	I-CNN (VGG19), D-CNN (VGG19)	CXR	Yonsei University, Korea	4000	random split	No	75354	Human reader
Aguiar et al, 2016	MLP	CXR	CFFH of the Federal University of Rio de Janeiro	216	Hold-out method	No	NR	Human reader
Faruk et al, 2021	Xception, InceptionV3, InceptionResNetV2, MobileNetV2	CT	Tuberculosis Chest X-ray Database	19600	Hold-out method	No	2800	Human reader
Karki et al, 2021	InceptionV3, Xception	CXR	TB Portals, MC, CH, TBX11K large scale tuberculosis datasetrtals dataset	11284	10-fold cross-validation	No	NR	Human reader
Dasanayaka et al, 2021	VGG16, InceptionV3, Ensemble	CXR	MC, CH, MIMIC database	6648	Hold-out method	No	831	Human reader
Shen et al, 2010	Bayesian classifier	CXR	Canada	18	NR	No	131	Human reader
Melendez et al, 2015	si-miSVM+PEDD	CXR	Zambia, Tanzania, the Gambia	1323	NR	No	1313	Human reader
Sharma et al, 2021	Tree, SVM, Naïve Bayes	CXR	India	2400	NR	No	600	Composite reference standard(s)
Arzhaeva et al, 2009	MVDB	CXR	NR (high population)	217	NR	No	165	Human reader
Jaeger et al, 2014	SVM	CXR	MC	138	NR	No	NR	Human reader
Chauhan et al, 2014	SVM	CXR	India	408	5-fold cross-validation	No	204	Human reader
Hogeweg et al, 2015	RF50, GB50, LDA, KNN13	CXR	Find & Treat, TB-NEAT	800	10-fold cross-validation	No	NR	MTB culture, human reader
Govindarajan et al, 2021	ELM, OSELM	CXR	MC	276	10-fold cross-validation	No	NR	Human reader
Acharya et al, 2022	ImageNet fine-tuned Normalization-Free Networks	CXR	Tuberculosis X-ray (TBX11K)	5880	Hold-out method	Yes	NR	Human reader
Kadry et al, 2022	VGG16, Fine Tree	CXR	IEEE	2100	Hold-out method	No	NR	Xpert MTB/RIF, Mycobacterium tuberculosis (MTB) culture, human reader
Kazemzadeh et al, 2023	NR	CXR	Europe, India, South Africa, Zambia, MC, CH	160187	Hold-out method	Yes	2309	Human reader
Margarat et al, 2022	DBN-AMBO	CXR	MC, CH	662	NR	No	NR	Human reader

Table S4. Accuracy measures reported by development studies.

Author and year	AUC	TP	FP	FN	TN	Sn	Sp	Accuracy	F1
Pasa et al, 2019	0.811(MC),<br>0.900(CH),<br>0.925(Belarus)	NR	NR	NR	NR	NR	NR	0.790(MC),<br>0.844(CH),<br>0.862(Belarus)	NR
Xie et al, 2020	0.977(MC),<br>0.941(CH),<br>0.993(FANXJU)	NR	NR	NR	NR	0.931(MC),<br>0.854(CH),<br>0.983(FANXJU)	0.923(MC),<br>0.951(CH),<br>0.962(FANXJU)	0.926(MC),<br>0.902(CH),<br>0.974(FANXJU)	NR
Ma et al, 2020	0.980	134	4	5	136	0.964	0.971	0.968	NR
Rajpurkar et al, 2020	NR	31	9	58	16	0.67	0.870	0.790	NR
Oloko-Oba et al, 2021	0.940(MC),<br>0.960(CH)	NR	NR	NR	NR	0.9813(MC),<br>0.9918(CH)	0.9578(MC),<br>0.9621(CH)	0.958(MC),<br>0.974(CH)	NR
Mamalakis et al, 2021	0.950	NR	NR	NR	NR	NR	NR	NR	0.758
Rajakumar et al, 2021	NR	587	20	21	585	0.9783	0.9667	0.973	NR
Wang et al, 2021	0.860,<br>0.780(validation)	NR	NR	NR	NR	0.92,<br>0.75(validation)	0.57,<br>0.63(validation)	0.830,<br>0.690(validation)	0.890,<br>0.700(validation)
Showkatian et al, 2022	0.870	NR	NR	NR	NR	0.87	NR	0.870	0.870
Zhou et al, 2022	0.998/0.56	NR	NR	NR	NR	0.798/0.844	0.996/0.934	0.948/0.931	NR
Rajaraman et al, 2021	0.954(CH),<br>0.964(MC)	NR	NR	NR	NR	0.8805(CH),<br>0.8772(MC)	0.8954(CH),<br>0.9687(MC)	0.888(CH),<br>0.923(MC)	0.887(CH),<br>0.919(MC)
Yan et al, 2021	NR	NR	NR	NR	NR	NR	NR	0.970(NH, YH),<br>0.954(NH, HH),<br>0.983(NH, NIH)	NR
Zhang et al, 2021	0.902	NR	NR	NR	NR	NR	NR	0.833	NR
Lakhani et al, 2017	0.980(AlexNet),<br>0.970(GoogLeNet)	71<br>75	4<br>1	6<br>6	69<br>69	0.920(AlexNet),<br>0.920(GoogLeNet)	0.947(AlexNet),<br>0.987(GoogLeNet)	0.933(AlexNet),<br>0.953(GoogLeNet)	NR
Han et al, 2021	0.965	NR	NR	NR	NR	0.96	0.971	NR	NR
An et al, 2022	NR	NR	NR	NR	NR	0.838	0.863	0.85	0.848
Lee et al, 2021	0.830, 0.840	NR	NR	NR	NR	NR	NR	NR	NR
Khatibi et al, 2021	0.990(MC),<br>0.980(CH)	NR	NR	NR	NR	NR	NR	0.993(MC),<br>0.992(CH)	NR
Kim et al, 2020	0.880,<br>0.910	NR<br>NR	NR<br>NR	NR<br>NR	NR<br>NR	0.85,<br>NR	0.76,<br>NR	NR<br>NR	NR<br>NR
Feng et al, 2020	0.809	NR	NR	NR	NR	0.908	0.608	0.828	NR
Hwang et al, 2019	0.988	NR	NR	NR	NR	0.980	0.980	NR	NR
Heo et al, 2019	0.908(I-CNN(VGG19)),<br>0.921(D-CNN(VGG19))	NR	NR	NR	NR	0.925(I-CNN(VGG19)),<br>0.925(D-CNN(VGG19))	NR	0.688(I-CNN(VGG19)),<br>0.748(D-CNN(VGG19))	NR
Aguiar et al, 2016	NR	56	23	2	189	0.96	0.89	NR	NR
Faruk et al, 2021	NR	687	13	5	695	NR (Xception),<br>NR (InceptionV3),<br>0.993(InceptionResNetV2),<br>NR(MobileNetV2)	NA (Xception),<br>NR (InceptionV3),<br>0.996(InceptionResNetV2),<br>NA(MobileNetV2)	0.960(Xception),<br>0.980(Inception V3),<br>0.994(InceptionResNetV2),<br>0.979(MobileNetV2)	0.950(Xception),<br>0.960(Inception V3),<br>0.990(InceptionResNetV2), 0.980(MobileNetV2)

Karki et al, 2021	0.810(InceptionV3),<br>0.810(Xception)	NR	NR	NR	NR	NR	NR	NR	NR
Dasanayaka et al, 2021	NR	139<br>143<br>144	9<br>6<br>5	8<br>4<br>3	121<br>124<br>125	0.945(VGG16),<br>0.972(InceptionV3),<br>0.979(Ensemble),	0.930(VGG16),<br>0.953(InceptionV3),<br>0.962(Ensemble),	0.938(VGG16),<br>0.963(InceptionV3),<br>0.971(Ensemble),	NR
Shen et al, 2010	NR	NR	NR	NR	NR	NR	NR	0.8235	NR
Melendez et al, 2015	0.860(Zambia),<br>0.860(Tanzania),<br>0.910(The Gambia)	NR	NR	NR	NR	NR	NR	NR	NR
Sharma et al, 2021	0.990(Tree),<br>0.957(SVM),<br>0.998(Naïve Bayes)	14<br>10<br>14	7<br>3<br>1	3<br>7<br>7	12<br>16<br>18	0.82(Tree),<br>0.58(SVM),<br>0.82(Naïve Bayes)	0.63(Tree),<br>0.84(SVM),<br>0.95(Naïve Bayes)	0.722(Tree),<br>0.722(SVM),<br>0.889(Naïve Bayes)	0.979(Tree),<br>0.800(SVM),<br>0.896(Naïve Bayes)
Arzhaeva et al, 2009	0.810	NR	NR	NR	NR	NR	NR	NR	NR
Jaeger et al, 2014	0.869	43	15	15	65	0.741	0.813	0.783	NR
Chauhan et al, 2014	0.942,<br>0.956,<br>0.860,<br>0.918	NR	NR	NR	NR	0.961,<br>0.885,<br>0.880,<br>0.920	0.923,<br>0.962,<br>0.840,<br>0.880	0.942,<br>0.923,<br>0.860,<br>0.900	0.943,<br>0.920,<br>0.863,<br>0.902
Hogeweg et al, 2015	0.868(RF50),<br>0.847(GB50),<br>0.741(LDA),<br>0.899(KNN13)	NR	NR	NR	NR	NR	NR	NR	NR
Govindarajan et al, 2021	NR<br>NR	57<br>56	0<br>1	1<br>2	80<br>79	0.993(ELM),<br>0.987(OSELM)	0.993(ELM),<br>0.987(OSELM)	0.993(ELM),<br>0.987(OSELM)	0.992(ELM),<br>0.986(OSELM)
Acharya et al, 2022	0.994	220	36	36	2244	0.918	0.984	0.969	NR
Kadry et al, 2022	NR	446	4	3	447	0.993	0.991	0.992	NR
Kazemzadeh et al, 2023	0.890	187	220	25	804	0.882	0.785	NR	NR
Margarat et al, 2022	NR	NR	NR	NR	NR	NR	0.991(CH)<br>0.994(MC)	0.992(CH)<br>0.987(MC)	NR

Abbreviation: CNN, convolutional neural networks; RCNN, regions with CNN features; CT, computed tomography; CXR, chest X-ray; TP, true positive; FP, false positive; FN, false negative; TN, true negative; MC, Montgomery Country; CH, Shenzhen Hospital, China; JSRT, Japanese Society of Radiology; FANXJU, First Affiliated Hospital of Xi'an Jiao Tong University; HIV, human immunodeficiency virus; ;NR, not reported; AUC, area under the receiver operating curve; Sn, sensitivity; Sp, specificity; MTB, Mycobacterium tuberculosis; NIH CXR, National Institute of Health ChestX-ray14 database; NIH CXR, National Institute of Health Chest X-ray15 database, NF, Nanfang Hospital; YH, yanling Hospital; HH, Haikou Hospital; CFFH, ClementiNo Fraga Filho Hospital; IEEE, Institute of Electrical and Electronics Engineers; DBN-AMBO, Deep Belief Network with Adaptive Monarch butterfly optimization.
