# Supplementary material for: Diagnostic Accuracy of the Artificial Intelligence Methods in Medical Imaging for Pulmonary Tuberculosis: A Systematic Review and Meta-Analysis
Source: J Clin Med. 2022 Dec 30;12(1):303. doi: 10.3390/jcm12010303 (PMC9820940; doi:10.3390/jcm12010303)
Supplement: Supplementary file 1 [file jcm-12-00303-s001.zip › Supplementary Materials File S2.pdf]

Supplementary Materials File S2

Embase <1974 to 2022 November> and Ovid MEDLINE(R) <1946 to November 2022> search through the OVID platform

Step	Term(s)
1	exp Tuberculosis/
2	tuberculosis.mp.
3	tuberculo*.mp.
4	tb.mp.
5	1 or 2 or 3 or 4
6	artificial intelligence.mp.
7	AI.mp.
8	machine learning.mp.
9	deep learning.mp.
10	ml.mp.
11	dl.mp.
12	exp "Neural Networks (Computer)"/
13	convolutional.mp.
14	cnn.mp.
15	computer-assisted.mp.
16	automated detection.mp.
17	automat*.mp.
18	(computer* adj3 diagnosis).mp.
19	(computer* adj2 detection).mp.
20	(computer* adj2 screening).mp.
21	6 or 7 or 8 or 9 or 10 or 11 or 12 or 13 or 14 or 15 or 16 or 17 or 18 or 19 or 20
22	exp Tomography, X-Ray Computed/
23	exp Radiography/
24	ct.mp.
25	computed tomography.mp.
26	xray.mp.
27	x-ray.mp.
28	CXR.mp.
29	radiograph*.mp.
30	photograph*.mp.
31	imag*.mp.
32	22 or 23 or 24 or 25 or 26 or 27 or 28 or 29 or 30 or 31
33	exp "Sensitivity and Specificity"/
34	exp roc curve/
35	area under the curve.mp.
36	auc.mp.

37	accurac*.mp.
38	diagnostic accuracy.mp.
39	specificity.mp.
40	sensitivity.mp.
41	roc.mp.
42	33 or 34 or 35 or 36 or 37 or 38 or 39 or 40 or 41
43	5 and 21 and 32 and 42
